# Neoadjuvant and Adjuvant Systemic Therapy for Newly Diagnosed Stage II–IV Epithelial Ovary, Fallopian Tube, or Primary Peritoneal Carcinoma: A Practice Guideline

**DOI:** 10.3390/curroncol29010022

**Published:** 2022-01-08

**Authors:** Hal Hirte, Raymond Poon, Xiaomei Yao, Taymaa May, Josee-Lyne Ethier, Lauri Petz, Jane Speakman, Laurie Elit

**Affiliations:** 1 Juravinski Cancer Centre, Division of Medical Oncology, McMaster University, Hamilton, ON L8V 5C2, Canada; 2 Program in Evidence-Based Care, Ontario Health (Cancer Care Ontario), Department of Oncology, McMaster University, Hamilton, ON L8V 1C3, Canada; yaoxia@mcmaster.ca; 3 Department of Health Research Methods, Evidence, and Impact, McMaster University, Hamilton, ON L8S 4L8, Canada; 4 Department of Obstetrics and Gynecology, Princess Margaret Hospital, University of Toronto, Toronto, ON M5G 2C1, Canada; Taymaa.May@uhn.ca; 5 Cancer Centre of Southeastern Ontario, Division of Cancer Care and Epidemiology, Cancer Research Institute, Department of Oncology and Medicine, Queen’s University, Kingston, ON K7L 3N6, Canada; josee-lyne.ethier@kingstonhsc.ca; 6 Independent Researcher, North Bay, ON P1B 8L7, Canada; northontgirl@hotmail.com; 7 Independent Researcher, Sutton, ON L0E 1R0, Canada; jspeakman2@me.com; 8 Juravinski Cancer Centre, Department of Obstetrics and Gynecology, McMaster University, Hamilton, ON L8V 5C2, Canada; elitlor@hhsc.ca

**Keywords:** ovarian cancer, neoadjuvant therapy, adjuvant therapy, intraperitoneal therapy, cytoreductive surgery, clinical practice guideline

## Abstract

Background: This study aims to provide guidance for the use of neoadjuvant and adjuvant systemic therapy in women with newly diagnosed stage II–IV epithelial ovary, fallopian tube, or primary peritoneal carcinoma. Methods: EMBASE, MEDLINE, and Cochrane Library were investigated for relevant systematic reviews and phase III trials. Articles focusing on consolidation and maintenance therapies were excluded. Results: For women with potentially resectable disease, primary cytoreductive surgery, followed by six to eight cycles of intravenous three-weekly paclitaxel and carboplatin is recommended. For those with a high-risk profile for primary cytoreductive surgery, neoadjuvant chemotherapy can be an option. Adjuvant chemotherapy with six cycles of dose-dense weekly paclitaxel plus three-weekly carboplatin can be considered for women of Japanese descent. In women with stage III or IV disease, the incorporation of bevacizumab concurrent with paclitaxel and carboplatin is not recommended for use as adjuvant therapy unless bevacizumab is continued as maintenance therapy. Intravenous paclitaxel plus intraperitoneal cisplatin and paclitaxel can be considered for stage III optimally debulked women who did not receive neoadjuvant chemotherapy. However, intraperitoneal administration of chemotherapy with bevacizumab should not be considered as an option for stage II–IV optimally debulked women. Discussion: The recommendations represent a current standard of care that is feasible to implement and valued by both clinicians and patients.

## 1. Introduction

Newly diagnosed ovarian cancer most commonly presents with disease that is already at an advanced stage. Once diagnosed, the main goal of treatment is to prevent or delay the recurrence of disease. Although ovarian cancer is a disease that generally responds well to chemotherapy, 5-year survivals decrease with increasing stage, 70% for stage II disease, but only 39% for stage III and 17% for stage IV [1]. Improving on this will require applying the available surgical and systemic therapy modalities in an optimal fashion. The optimal timing of surgery, whether upfront or after initial neoadjuvant chemotherapy, remains controversial. Additionally, various administration schedules of chemotherapy have included intraperitoneal (i.p.) delivery and dose-dense regimens. Thus, the Working Group of the Ovarian Cancer Guideline Development Group, in association with the Program in Evidence-Based Care (PEBC) of Ontario Health (Cancer Care Ontario), intended to develop this clinical practice guideline to make recommendations on the most effective regimen to administer systemic therapy for women with newly diagnosed stage II, III or IV epithelial ovary, fallopian tube, or primary peritoneal carcinoma (EOC) in the neoadjuvant/adjuvant setting. This process includes a systematic review, interpretation of the evidence and draft recommendations by the Working Group, internal review by content and methodology experts, and external review by Ontario clinicians and other stakeholders. This guideline did not include the role consolidation or maintenance therapies.

## 2. Material and Methods

### 2.1. Research Questions

(i) What is the optimal regimen (dose/schedule/frequency) for women who will receive neoadjuvant therapy before interval cytoreduction or adjuvant therapy after primary cytoreduction?

(ii) What is the optimal regimen (dose/schedule/frequency) and most effective mode of administration (intravenous (i.v.) versus i.p.) for optimally debulked women (<1 cm residual disease) who will receive adjuvant therapy?

(iii) Do women with BRCA mutation, different histological subtypes (low-grade serous, endometrioid, clear cell, mucinous, undifferentiated or unclassifiable), location subtypes, residual disease after cytoreduction, or stage receiving neoadjuvant, or adjuvant therapy have different optimal regimen (dose/schedule/frequency) and outcomes?

### 2.2. Literature Search

A search for relevant systematic reviews and Phase III randomized-controlled trials was conducted in MEDLINE, EMBASE, and Cochrane Library from January 2003 to October 2019. Furthermore, conference abstracts were searched from 2017 to 2019 and full publications of included abstracts were later retrieved via PubMed. Briefly, trials that investigated chemotherapy, targeted therapy, immunotherapy, or hormonal therapy were deemed eligible for inclusion. Articles on consolidation and maintenance therapies were excluded. A detailed description of the methods can be found in our systematic review [2].

### 2.3. Internal Review

The Ovarian Cancer Guideline Development Group is comprised of content experts in Ontario who will vote via email to indicate whether they (1) approve the document without further comment or with the following comments and changes; (2) disapprove the document due to the following reasons; or (3) abstain from voting for any specified reason (e.g., the guideline is not in my expertise). For document approval, 75% must cast a vote to approve. In addition, the document must be unanimously approved by the PEBC Report Approval Panel (RAP), which is a panel consisting of three reviewers with methodology expertise. The Expert Panel and RAP members may specify that approval is conditional, and that changes to the document are required.

### 2.4. External Review

Content experts and target users provide feedback on the approved draft guideline through two processes, Targeted Peer Review and Professional Consultation. First, the Working Group identified several individuals with content expertise and asked them to review and provide feedback on the guideline document. Secondly, potential guideline users and relevant care providers are asked to complete a brief online survey that allows them to provide guideline recommendation feedback.

## 3. Results

### 3.1. Literature Search Results

The literature search and the systematic review details were provided in our systematic review [2]. Overall, 33 phase III trials were included to support our recommendations below.

### 3.2. Internal Review

#### 3.2.1. Expert Panel Review and Approval

Of the eight members of the Ovarian Cancer Guideline Development Group, seven members voted and one abstained, for a total of 87.5% response in August 2020. Of those who voted, seven approved the document (100%). Summary of the Working Group’s responses to comments from the Ovarian Cancer Guideline Development Group are as follows: (1) The international recommendation for achieving optimal cytoreduction is no residual disease. [Response: This remains controversial as optimal is still considered ≤1 cm; however, “ideally to no visible disease” was added to the qualifying statement for recommendation 1]; (2) Poor performance status should be defined according to the Eastern Cooperative Oncology Group. [Response: A definition was added to the qualifying statement for recommendation 1]; (3) If the alternative to paclitaxel and carboplatin is offered, need to add the dose adjustment for carboplatin with docetaxel as per SCOTROC 1 trial [3]. [Response: The dose adjustment for carboplatin was added to the qualifying statement for recommendation 2]; (4) There should be an additional statement about the role of additional maintenance treatments (bevacizumab, poly ADP ribose polymerase [PARP]) for certain subgroups. [Response: Maintenance therapies are specifically addressed in a separate guideline (An Ontario Health (Cancer Care Ontario) Clinical Practice Guideline: Consolidation or Maintenance Systemic Therapy for Newly Diagnosed stage II, III, or IV Epithelial Ovary, Fallopian Tube, or Primary Peritoneal Carcinoma [4]); (5) “not from Japan” should be modified to Japanese heritage or descent or ethnicity. [Response: The qualifying statement for recommendation 2 was modified to “Japanese descent”]; (6) Need to specify that bevacizumab has no benefit with adjuvant chemotherapy if it is not continued, but it has benefit when it is started with adjuvant chemotherapy and continued as maintenance for high-risk. This is important to highlight as bevacizumab needs to be started with adjuvant chemotherapy and not only as maintenance or with the last cycle of chemotherapy. [Response: The wording of recommendation 4 was modified to provide more clarity. A qualifying statement was added to support the use of bevacizumab as adjuvant therapy concurrent with paclitaxel and carboplatin and continued as maintenance therapy in high-risk disease women]; (7) “is recommended” should be changed to “can be considered” since debate around intraperitoneal chemotherapy persists as well as the negative results from GOG 252 [5]. [Response: Recommendation 5 was modified to “can be considered”]; (8) The hyperthermic intraperitoneal chemotherapy (HIPEC) option should be better addressed. There is strong evidence from the Willemien J. van Driel study [6] for overall survival (OS) benefit of 12 months in stage III disease patients who had neoadjuvant chemotherapy and HIPEC at the time of interval cytoreductive surgery versus patients who had only interval cytoreductive surgery without HIPEC. This should be added to the treatment options for this group of patients. [Response: This is beyond the scope for this review].

#### 3.2.2. RAP Review and Approval

Three RAP members, including the Scientific Director of PEBC, reviewed and approved this document in August 2020. The following is a summary of the Working Group’s responses to comments from RAP: (1) The qualifying statement for recommendation 1 is vague, consider adding one or two examples of the most common significant symptoms. [Response: A few examples of the most common significant disease-related symptoms have been added]; (2) It is unclear as to why the use of neoadjuvant chemotherapy is a weak recommendation in comparison to a regular recommendation for adjuvant therapy. It appears the studies were fairly rigorous, the data strong, and there seems not to be a detrimental effect of neoadjuvant chemotherapy on surgical outcomes. [Response: A point was added to the justification for recommendation 1 to explain the rationale for a weak recommendation].

### 3.3. External Review

#### 3.3.1. Targeted Peer Review

Six targeted peer reviewers from Ontario, British Columbia and Quebec who are considered to be clinical and/or methodological experts on the topic were identified by the Working Group. Three agreed to be the reviewers and their responses were received. The following is a summary of the Working Group’s responses to comments from targeted peer reviewers: (1) The Working Group is very small so personal bias will affect the strength of recommendations, specifically relevant to the neoadjuvant chemotherapy question where there is bias of surgeons towards upfront surgery. This small size of the Working Group is a major problem with the paper as inevitably it will lead to it being a more personal opinion than a true reflection of what the majority of those who practice in this area believe. [Response: In addition to the Working Group (which included one medical oncologist, three gynecologic oncologists, two guideline methodologists, and two patient representatives), an expert panel comprised of a diverse group of seven clinicians, as well as a three-person panel with methodology expertise reviewed and approved the recommendations]; (2) Caelyx can replace Carbo-Taxol with equivalent efficacy. [Response: In the MITO-2 trial [7] with a median follow-up of 40 months, carboplatin plus pegylated liposomal doxorubicin did not provide a significant survival advantage over carboplatin plus paclitaxel and led to more grade 3 or 4 anemia (10.1% versus 3.7%, *p* = 0.0003) and thrombocytopenia (15.9% versus 2.0%, *p* < 0.01) but less neurotoxicity (0.3% versus 2.9%, *p* = 0.0035)]; (3) According to GOG 172 [8], paclitaxel is given as 135 mg/m^2^ over 24 h with cisplatin. On the other hand, paclitaxel is given as 175 mg/m^2^ over 3 h with carboplatin. [Response: Information regarding infusion duration was added to recommendations 1, 2 and 5]; (4) The evaluation by a gynecologic oncologist for surgical eligibility (primary surgery versus interval cytoreduction) should be substantiated by reference. [Response: The criteria used to identify women who are not suitable for primary cytoreductive surgery were based on expert consensus from the Working Group and the Ovarian Cancer Guideline Development Group]; (5) Discussion on histological heterogeneity with regard to the choice of treatment should be included. [Response: Subgroup analysis based on histological subtypes did not favor one regimen over the other. Further research is required to provide treatment guidance for different histological types or molecular subsets in the target population]; (6) Discussion on BRCA-HRD status as part of the decision-making assessment of the whole therapeutic strategy should be included. [Response: Only one post hoc analysis examined the prognostic relevance of BRCA1 expression [9]. In brief, women with aberrant BRCA1 expression had increased OS when treated with i.p. chemotherapy. Further research is required to investigate BRCA-HRD status as part of treatment decision making in the target population]; (7) Please justify recommending up to eight cycles of carboplatin and paclitaxel based on the literature. [Response: Despite the majority of the trials administering six cycles of carboplatin and paclitaxel, there were four trials (JCOG 0602 [10], OV16 [11], GOG 0182-ICON5 [12], and NSGO-EORTC GCG-NIC CTG [13]) that included up to eight cycles in their study arms. Therefore, six cycles is the standard but one could use up to eight and be within the parameters of prior trials].

#### 3.3.2. Professional Consultation

Feedback was obtained through a brief online survey of healthcare professionals and other stakeholders who are the intended users of the guideline. All the gynecologic oncologists and medical oncologists with an interest in ovarian cancer in the PEBC database were contacted by email to inform them of the survey. A total of 110 professionals were contacted, all of which practice in Ontario. Sixteen (14.5%) responses were received. Eight stated that they did not have interest in this area or were unavailable to review this guideline at the time, and one stated they were now retired. The following is a summary of the Working Group’s responses to comments from professional consultants: (1) Most gynecologic oncologists feel that optimal debulking is preferable to neoadjuvant chemotherapy. It is an issue of feasibility, surgeon skills, and decision making. This are hard to enunciate in a written document. Some surgeons are very aggressive, some not at all, and most in between. [Response: This is certainly a valid point].

## 4. Clinical Practice Guideline

The definition for the strength of recommendations implemented in this guideline is listed in Table 1.

### 4.1. Recommendation 1

For women with stage III or IV EOC who may have a high-risk profile for primary cytoreductive surgery as determined by a gynecologic oncologist, neoadjuvant chemotherapy with three to four cycles of intravenous (i.v.) three-weekly paclitaxel (175 mg/m^2^ over 3 h) and carboplatin (area under the curve [AUC] = 5/6), then interval cytoreductive surgery, followed in turn by three to four cycles of i.v. three-weekly paclitaxel (175 mg/m^2^ over 3 h) and carboplatin (AUC = 5/6) can be recommended as an option (Strength: weak recommendation).

#### 4.1.1. Qualifying Statement

High risk is defined as significant disease related symptoms (e.g., moderate to severe pleural effusion, cachexia with poor oral intake, hypoalbuminemia and other poor nutritional status), low likelihood of achieving optimal cytoreduction (residual ≤ 1 cm, but ideally to no visible disease), or poor prognostic factors (e.g., poor performance status [PS] according to the Eastern Cooperative Oncology Group, PS > 2).

#### 4.1.2. Key Evidence

Three trials (EORTC 55971, CHORUS, and JCOG 0602) used a non-inferiority design [10,14,15,16,17] and one used a superiority design (SCORPION) [18,19] to compare upfront primary debulking surgery (followed by at least six cycles of carboplatin or cisplatin plus paclitaxel) to neoadjuvant chemotherapy (three to four cycles before and three to four cycles after interval debulking surgery). A detailed description of the key evidence can be found in [2].

#### 4.1.3. Justification

Neoadjuvant chemotherapy is associated with lower postoperative mortality and a general trend toward fewer adverse events and higher QoL scores then primary cytoreductive surgery followed by adjuvant therapy. Despite the two earlier trials (EORTC 55971 and CHORUS) showing that OS was non-inferior to that of primary cytoreductive surgery, the more recent trial (JCOG 0602) was unable to corroborate the non-inferiority of neoadjuvant chemotherapy. Additionally, the SCORPION trial failed to show superiority with respect to PFS for neoadjuvant chemotherapy.

The JCOG 0602 trial administered up to eight cycles of paclitaxel and carboplatin in their study arms; however, there is no direct evidence comparing six cycles to more than six cycles of chemotherapy. Despite six cycles of paclitaxel and carboplatin (three before and three after interval debulking surgery) being by and large the standard, the Working Group members will defer to the end users to make their own decision based on individual clinical situation. Furthermore, the Working Group members consider the criteria used to determine a high-risk profile both acceptable and feasible in current practice.

### 4.2. Recommendation 2

For women with stage II, III, or IV EOC and potentially resectable disease as determined by a gynecologic oncologist, primary cytoreductive surgery, followed by six to eight cycles of i.v. three-weekly paclitaxel (175 mg/m^2^ over 3 h) and carboplatin (AUC = 5/6) is recommended (Strength: strong recommendation).

#### 4.2.1. Qualifying Statements

For those who are unable to tolerate paclitaxel, an alternate regimen consisting of docetaxel (75 mg/m^2^) may be offered with carboplatin (AUC = 5). Adjuvant chemotherapy with six cycles of dose-dense weekly paclitaxel (80 mg/m^2^) in combination with three-weekly carboplatin (AUC = 6) administered intravenously can be considered for women with stage II, III, or IV EOC of Japanese descent.

#### 4.2.2. Key Evidence

Six trials [3,7,11,12,20,21] compared the efficacy of various platinum-based doublet regimens against standard paclitaxel and carboplatin, while four trials [22,23,24,25,26,27,28] compared a dose-dense weekly regimen against a standard three-weekly schedule. A detailed description of the key evidence can be found in [2].

#### 4.2.3. Justification

The three-weekly regimen consisting of paclitaxel and carboplatin remains the standard of care. For those women who cannot tolerate paclitaxel, the Working Group members consider docetaxel as an alternative, owing to its similar efficacy in terms of PFS, while reducing the likelihood of neurotoxicity and improving the level of treatment-related QoL. Docetaxel is also less likely to induce hypersensitivity reactions. Again, both the OV16 and GOG 0182-ICON5 trials administered up to eight cycles of paclitaxel and carboplatin in their study arms and the Working Group members will defer to the end users to make their own decision regarding optimal treatment duration based on the individual clinical situation.

Although weekly paclitaxel can improve PFS and OS according to JGOG 3016, 36.2% of women discontinued this regimen prematurely due to toxic effects compared with 21.6% in the conventional regimen group. Since the trial enrolled only women living in Japan, there may exist pharmacogenomics differences between the Japanese and non-Japanese populations, which limits the generalizability of these results. Considering the uncertainty of the evidence and the unfavorable results from ICON8, the Working Group members could not make a recommendation for a dose-dense weekly regimen over a standard three-weekly schedule for the general population. However, it is important to keep in mind that the differences in hematological toxicity for the dose-dense weekly regimen may simply be due to more frequent testing.

In the GOG 0262 trial, the small subset of women (16% in each treatment group) who opted not to receive bevacizumab with weekly paclitaxel saw an improvement in PFS. However, OS was not analyzed while adverse events and QoL scores were not reported separately from those who received bevacizumab. Thus, there is no evidence for the Working Group members to support adding bevacizumab into adjuvant therapy.

### 4.3. Recommendation 3

The addition of a third chemotherapy agent to standard paclitaxel and carboplatin is not recommended for use as adjuvant therapy in women with stage II, III, or IV EOC (Strength: strong recommendation).

#### 4.3.1. Key Evidence

The efficacy of adding a third chemotherapy agent to a standard paclitaxel and carboplatin regimen was examined in six trials [12,13,29,30,31,32]. A detailed description of the key evidence can be found in [2].

#### 4.3.2. Justification

The incorporation of a third chemotherapy drug to paclitaxel and carboplatin has not been shown to improve OS and PFS. Given the absence of a survival benefit along with increased toxicity, the Working Group members recommend not to use platinum-based triplet chemotherapy in women with stage II, III, or IV EOC.

### 4.4. Recommendation 4

The incorporation of bevacizumab concurrent with paclitaxel and carboplatin is not recommended for use as adjuvant therapy unless bevacizumab is continued as maintenance therapy in women with stage III or IV EOC (Strength: strong recommendation).

#### 4.4.1. Qualifying Statement

Concurrent use of i.v. three-weekly bevacizumab (7.5 mg/kg) with paclitaxel and carboplatin for six cycles and continued for up to 12 cycles or until progression as maintenance therapy can be recommended for women with newly diagnosed high-risk stage III (residual disease > 1 cm or inoperable), or stage IV EOC.

#### 4.4.2. Key Evidence

The efficacy of adding a targeted agent to a standard paclitaxel and carboplatin regimen was examined in one trial [33,34,35]. A detailed description of the key evidence can be found in [2].

#### 4.4.3. Justification

The incorporation of bevacizumab (without continued treatment as maintenance) to paclitaxel and carboplatin resulted in increased toxicity and no improvement in survival. Hence, the Working Group members do not recommend bevacizumab as adjuvant therapy for women with stage III or IV EOC. However, high-risk women, such as those with sub-optimally debulked stage III disease (residual disease > 1 cm), inoperable stage III, or stage IV disease, appeared to benefit the most with the incorporation of bevacizumab concurrent with chemotherapy and continued as maintenance [36,37,38]. A similar case could be made for advocating for the concurrent use of veliparib with adjuvant therapy and continued as maintenance in stage III or IV EOC with homologous-recombination deficiency [39]. Please refer to a separate guideline on consolidation/maintenance systemic therapy [4].

### 4.5. Recommendations 5 and 6

i.v. paclitaxel (135 mg/m^2^ over 24 h) plus intraperitoneal (i.p.) cisplatin (100 mg/m^2^) and paclitaxel (60 mg/m^2^) can be considered for stage III optimally debulked women (≤1 cm residual disease) who did not receive neoadjuvant chemotherapy (Strength: weak recommendation).

i.p. administration of chemotherapy with bevacizumab should not be considered as an option for stage II to IV optimally debulked women (≤1 cm residual disease) (Strength: strong recommendation).

#### 4.5.1. Key Evidence

Two trials (GOG 172 and GOG 252) [5,8,9,40] compared i.p. chemotherapy versus conventional i.v. chemotherapy. A detailed description of the key evidence can be found in [2].

#### 4.5.2. Justification

Given the results of the GOG 172 trial, the Working Group members determined that the substantial increase in OS and PFS conferred by i.v. paclitaxel plus i.p. cisplatin and paclitaxel outweigh the associated adverse events and lower patient reported QoL scores. Furthermore, pathogenic BRCA mutations are more common than expected in optimally resected ovarian cancer patients selected for IP therapy. IP therapy was associated with a dramatic improvement in PFS and OS in BRCA+ patients compared with BRCA- patients. This improvement is greater than has been reported for BRCA+ patients with IV chemotherapy. The magnitude of this benefit suggests that patients with pathogenic mutations in BRCA may benefit from IP therapy [41].

In the GOG 252 trial, both regimens consisting of i.p. chemotherapy plus bevacizumab offered no survival benefit and some harms in terms of toxicity and QoL. Thus, the Working Group members would not consider this as an acceptable treatment option.

## 5. Discussion

This evidence-based clinical practice guideline included four strong recommendations and two weak recommendations regarding the role of neoadjuvant and adjuvant systemic therapy (plus the addition of bevacizumab) for newly diagnosed stage II–IV epithelial ovary, fallopian tube, or primary peritoneal carcinoma (Table 2). These recommendations represent the current standard of care that is feasible to implement and valued by both clinicians and patients. The additional role of consolidation or maintenance therapy with other agents was not part of this guideline, as those were addressed in a previous guideline [4]. Subgroup analysis based on histological subtypes [42] did not favor one regimen over the other. Further research is required to investigate different histological types or BRCA-HRD status as part of treatment decision making in the neoadjuvant and adjuvant settings. The pending results of the iPocc study will clarify the role of intraperitoneal chemotherapy, if any, in both optimally and sub optimally debulked women.

## Figures and Tables

**Table 1:** Strength of recommendations for this guideline. The factors considered in the below judgments include desirable and undesirable effects of the neoadjuvant and adjuvant therapy, the certainty of evidence, patient preference, health equity, acceptability, feasibility, and generalizability.

Strength of Recommendations for This Guideline	Definition
Strong recommendation to use the intervention	The guideline Working Group * believes the benefits of the neoadjuvant or adjuvant therapy in newly diagnosed stage II, III, or IV ovarian cancer patients clearly outweigh the harms for nearly all patients and the group is confident to support the recommended action.
Weak recommendation to use the intervention	The guideline Working Group * believes the benefits and harms of the neoadjuvant or adjuvant therapy in the target patients are closely balanced or are more uncertain but still adequate to support the recommended action.
No recommendation for the intervention	The guideline Working Group * is uncertain whether the benefits and harms of the neoadjuvant or adjuvant therapy in the target patients are balanced and does not recommend a specific action.
Weak recommendation not to use the intervention	The guideline Working Group * believes the benefits and harms of the neoadjuvant or adjuvant therapy in the target patients are closely balanced or are more uncertain but still adequate to support the recommended action.
Strong recommendation not to use the intervention	The guideline Working Group * believes the harms of the neoadjuvant or adjuvant therapy in the target patients clearly outweigh the benefits for nearly all patients and the group is confident to support the recommended action.

* The guideline Working Group includes one medical oncologist, three gynecologic oncologists, two guideline methodologists, and two patient representatives.

**Table 2:** Summary of Recommendations.

Recommendation	Strength of Recommendation
For women with stage III or IV EOC who may have a high-risk profile for primary cytoreductive surgery as determined by a gynecologic oncologist, neoadjuvant chemotherapy with three to four cycles of intravenous (i.v.) three-weekly paclitaxel (175 mg/m^2^ over 3 h) and carboplatin (area under the curve [AUC] = 5/6), then interval cytoreductive surgery, followed in turn by three to four cycles of i.v. three-weekly paclitaxel (175 mg/m^2^ over 3 h) and carboplatin (AUC = 5/6) can be recommended as an option	Weak
For women with stage II, III, or IV EOC and potentially resectable disease as determined by a gynecologic oncologist, primary cytoreductive surgery, followed by six to eight cycles of i.v. three-weekly paclitaxel (175 mg/m^2^ over 3 h) and carboplatin (AUC = 5/6) is recommended	Strong
The addition of a third chemotherapy agent to standard paclitaxel and carboplatin is not recommended for use as adjuvant therapy in women with stage II, III, or IV EOC	Strong
The incorporation of bevacizumab concurrent with paclitaxel and carboplatin is not recommended for use as adjuvant therapy unless bevacizumab is continued as maintenance therapy in women with stage III or IV EOC	Strong
i.v. paclitaxel (135 mg/m^2^ over 24 h) plus intraperitoneal (i.p.) cisplatin (100 mg/m^2^) and paclitaxel (60 mg/m^2^) can be considered for stage III optimally debulked women (≤1 cm residual disease) who did not receive neoadjuvant chemotherapy	Weak
i.p. administration of chemotherapy with bevacizumab should not be considered as an option for stage II to IV optimally debulked women (≤1 cm residual disease)	Strong

## Data Availability

Not applicable.

## References

[1] Heintz A.P.M., Odicino F., Maisonneuve P., Quinn M.A., Benedet J.L., Creasman W.T., Ngan H.Y.S., Pecorelli S., Beller U. (2006). Carcinoma of the Ovary. Int. J. Gynecol. Obstet..

[2] Hirte H., Poon R., Yao X., May T., Ethier J.L., Petz L., Speakman J., Elit L. (2021). Neoadjuvant and adjuvant systemic therapy for newly diagnosed stage II–IV epithelial ovary, fallopian tube, or primary peritoneal carcinoma: A systematic review. Crit. Rev. Oncol. Hematol..

[3] Vasey P.A., Jayson G.C., Gordon A., Gabra H., Coleman R., Atkinson R., Parkin D., Paul J., Hay A., Kaye S.B. (2004). Phase III randomized trial of docetaxel-carboplatin versus paclitaxel-carboplatin as first-line chemotherapy for ovarian carcinoma. J. Natl. Cancer Inst..

[4] Hirte H., Yao X., Ferguson S.E., May T., Elit L. (2021). An Ontario Health (Cancer Care Ontario) Clinical Practice Guideline: Consolidation or Maintenance Systemic Therapy for Newly Diagnosed Stage II, III, or IV Epithelial Ovary, Fallopian Tube, or Primary Peritoneal Carcinoma. Curr. Oncol..

[5] Walker J.L., Brady M.F., Wenzel L., Fleming G.F., Huang H.Q., DiSilvestro P.A., Fujiwara K., Alberts D.S., Zheng W., Tewari K.S. (2019). Randomized Trial of Intravenous Versus Intraperitoneal Chemotherapy Plus Bevacizumab in Advanced Ovarian Carcinoma: An NRG Oncology/Gynecologic Oncology Group Study. J. Clin. Oncol..

[6] van Driel W.J., Koole S.N., Sikorska K., van Schagen Leeuwen J.H., Schreuder H.W.R., Hermans R.H.M., de Hingh I.H.J.T., van der Velden J., Arts H.J., Massuger L.F.A.G. (2018). Hyperthermic Intraperitoneal Chemotherapy in Ovarian Cancer. N. Engl. J. Med..

[7] Pignata S., Scambia G., Ferrandina G., Savarese A., Sorio R., Breda E., Gebbia V., Musso P., Frigerio L., Del Medico P. (2011). Carboplatin plus paclitaxel versus carboplatin plus pegylated liposomal doxorubicin as first-line treatment for patients with ovarian cancer: The MITO-2 randomized phase III trial. J. Clin. Oncol..

[8] Armstrong D.K., Bundy B., Wenzel L., Huang H.Q., Baergen R., Lele S., Copeland L.J., Walker J.L., Burger R.A., Mackey D. (2006). Intraperitoneal cisplatin and paclitaxel in ovarian cancer. N. Engl. J. Med..

[9] Lesnock J.L., Darcy K.M., Tian C., Deloia J.A., Thrall M.M., Zahn C., Armstrong D.K., Birrer M.J., Krivak T.C. (2013). BRCA1 expression and improved survival in ovarian cancer patients treated with intraperitoneal cisplatin and paclitaxel: A Gynecologic Oncology Group Study. Br. J. Cancer.

[10] Onda T., Satoh T., Ogawa G., Saito T., Kasamatsu T., Nakanishi T., Mizutani T., Takehara K., Okamoto A., Ushijima K. (2020). Comparison of survival between primary debulking surgery and neoadjuvant chemotherapy for stage III/IV ovarian, tubal and peritoneal cancers in phase III randomised trial. Eur. J. Cancer.

[11] Hoskins P., Vergote I., Cervantes A., Tu D., Stuart G., Zola P., Poveda A., Provencher D., Katsaros D., Ojeda B. (2010). Advanced ovarian cancer: Phase III randomized study of sequential cisplatin-topotecan and carboplatin-paclitaxel vs carboplatin-paclitaxel. J. Natl. Cancer Inst..

[12] Bookman M.A., Brady M.F., McGuire W.P., Harper P.G., Alberts D.S., Friedlander M., Colombo N., Fowler J.M., Argenta P.A., De Geest K. (2009). Evaluation of new platinum-based treatment regimens in advanced-stage ovarian cancer: A phase III trial of the gynecologic cancer intergroup. J. Clin. Oncol..

[13] Lindemann K., Christensen R.D., Vergote I., Stuart G., Izquierdo M.A., Kaern J., Havsteen H., Eisenhauer E., Ridderheim M., Lopez A.B. (2012). First-line treatment of advanced ovarian cancer with paclitaxel/carboplatin with or without epirubicin (TEC versus TC)-a gynecologic cancer intergroup study of the NSGO, EORTC GCG and NCIC CTG. Ann. Oncol..

[14] Vergote I., Trope C.G., Amant F., Kristensen G.B., Ehlen T., Johnson N., Verheijen R.H.M., Van Der Burg M.E.L., Lacave A.J., Panici P.B. (2010). Neoadjuvant chemotherapy or primary surgery in stage IIIC or IV ovarian cancer. N. Engl. J. Med..

[15] Greimel E., Kristensen G.B., Van Der Burg M.E.L., Coronado P., Rustin G., Del Rio A.S., Reed N.S., Nordal R.R., Coens C., Vergote I. (2013). Quality of life of advanced ovarian cancer patients in the randomized phase III study comparing primary debulking surgery versus neo-adjuvant chemotherapy. Gynecol. Oncol..

[16] Kehoe S., Hook J., Nankivell M., Jayson G.C., Kitchener H., Lopes T., Luesley D., Perren T., Bannoo S., Mascarenhas M. (2015). Primary chemotherapy versus primary surgery for newly diagnosed advanced ovarian cancer (CHORUS): An open-label, randomised, controlled, non-inferiority trial. Lancet.

[17] Onda T., Satoh T., Saito T., Kasamatsu T., Nakanishi T., Nakamura K., Wakabayashi M., Takehara K., Saito M., Ushijima K. (2016). Comparison of treatment invasiveness between upfront debulking surgery versus interval debulking surgery following neoadjuvant chemotherapy for stage III/IV ovarian, tubal, and peritoneal cancers in a phase III randomised trial: Japan Clinical Oncology Group Study JCOG0602. Eur. J. Cancer.

[18] Fagotti A., Ferrandina M.G., Vizzielli G., Pasciuto T., Fanfani F., Gallotta V., Margariti P.A., Chiantera V., Costantini B., Gueli Alletti S. (2020). Randomized trial of primary debulking surgery versus neoadjuvant chemotherapy for advanced epithelial ovarian cancer (SCORPION-NCT01461850). Int. J. Gynecol. Cancer.

[19] Fagotti A., Ferrandina G., Vizzielli G., Fanfani F., Gallotta V., Chiantera V., Costantini B., Margariti P.A., Gueli Alletti S., Cosentino F. (2016). Phase III randomised clinical trial comparing primary surgery versus neoadjuvant chemotherapy in advanced epithelial ovarian cancer with high tumour load (SCORPION trial): Final analysis of peri-operative outcome. Eur. J. Cancer.

[20] Aravantinos G., Fountzilas G., Kosmidis P., Dimopoulos M.A., Stathopoulos G.P., Pavlidis N., Bafaloukos D., Papadimitriou C., Karpathios S., Georgoulias V. (2005). Paclitaxel plus carboplatin versus paclitaxel plus alternating carboplatin and cisplatin for initial treatment of advanced ovarian cancer: Long-term efficacy results: A Hellenic Cooperative Oncology Group (HeCOG) study. Ann. Oncol..

[21] Li L., Zhuang Q., Cao Z., Yin R., Zhu Y., Zhu L., Xie X., Zhang Y., Li L., Wu Q. (2018). Paclitaxel plus nedaplatin vs. paclitaxel plus carboplatin in women with epithelial ovarian cancer: A multi-center, randomized, open-label, phase III trial. Oncol. Lett..

[22] Chan J.K., Brady M.F., Penson R.T., Huang H., Birrer M.J., Walker J.L., DiSilvestro P.A., Rubin S.C., Martin L.P., Davidson S.A. (2016). Weekly vs. every-3-week paclitaxel and carboplatin for ovarian cancer. N. Engl. J. Med..

[23] Katsumata N., Yasuda M., Isonishi S., Takahashi F., Michimae H., Kimura E., Aoki D., Jobo T., Kodama S., Terauchi F. (2013). Long-term results of dose-dense paclitaxel and carboplatin versus conventional paclitaxel and carboplatin for treatment of advanced epithelial ovarian, fallopian tube, or primary peritoneal cancer (JGOG 3016): A randomised, controlled, open-label trial. Lancet Oncol..

[24] Clamp A.R., James E.C., McNeish I.A., Dean A., Kim J.-W., O’Donnell D.M., Hook J., Coyle C., Blagden S., Brenton J.D. (2019). Weekly dose-dense chemotherapy in first-line epithelial ovarian, fallopian tube, or primary peritoneal carcinoma treatment (ICON8): Primary progression free survival analysis results from a GCIG phase 3 randomised controlled trial. Lancet.

[25] Katsumata N., Yasuda M., Takahashi F., Isonishi S., Jobo T., Aoki D., Tsuda H., Sugiyama T., Kodama S., Kimura E. (2009). Dose-dense paclitaxel once a week in combination with carboplatin every 3 weeks for advanced ovarian cancer: A phase 3, open-label, randomised controlled trial. Lancet.

[26] Harano K., Terauchi F., Katsumata N., Takahashi F., Yasuda M., Takakura S., Takano M., Yamamoto Y., Sugiyama T. (2014). Quality-of-life outcomes from a randomized phase III trial of dose-dense weekly paclitaxel and carboplatin compared with conventional paclitaxel and carboplatin as a first-line treatment for stage II-IV ovarian cancer: Japanese Gynecologic Oncology Group trial (JGOG3016). Ann. Oncol..

[27] Blagden S.P., Cook A.D., Poole C., Howells L., McNeish I.A., Dean A., Kim J.-W., O’Donnell D.M., Hook J., James E.C. (2020). Weekly platinum-based chemotherapy versus 3-weekly platinum-based chemotherapy for newly diagnosed ovarian cancer (ICON8): Quality-of-life results of a phase 3, randomised, controlled trial. Lancet Oncol..

[28] Pignata S., Scambia G., Katsaros D., Gallo C., Pujade-Lauraine E., De Placido S., Bologna A., Weber B., Raspagliesi F., Panici P.B. (2014). Carboplatin plus paclitaxel once a week versus every 3 weeks in patients with advanced ovarian cancer (MITO-7): A randomised, multicentre, open-label, phase 3 trial. Lancet Oncol..

[29] Aravantinos G., Fountzilas G., Bamias A., Grimani I., Rizos S., Kalofonos H.P., Skarlos D.V., Economopoulos T., Kosmidis P.A., Stathopoulos G.P. (2008). Carboplatin and Paclitaxel versus Cisplatin, Paclitaxel and Doxorubicin for first-line chemotherapy of Advanced Ovarian Cancer: A Hellenic Cooperative Oncology Group (HeCOG) study. Eur. J. Cancer.

[30] Du Bois A., Weber B., Rochon J., Meier W., Goupil A., Olbricht S., Barats J.C., Kuhn W., Orfeuvre H., Wagner U. (2006). Addition of epirubicin as a third drug to carboplatin-paclitaxel in first-line treatment of advanced ovarian cancer: A prospectively randomized Gynecologic Cancer Intergroup trial by the Arbeitsgemeinschaft Gynaekologische Onkologie Ovarian Cancer Study Group and the Groupe d’Investigateurs Nationaux pour l’Etude des Cancers Ovariens. J. Clin. Oncol..

[31] Du Bois A., Herrstedt J., Hardy-Bessard A.C., Muller H.H., Harter P., Kristensen G., Joly F., Huober J., Avall-Lundqvist E., Weber B. (2010). Phase III trial of carboplatin plus paclitaxel with or without gemcitabine in first-line treatment of epithelial ovarian cancer. J. Clin. Oncol..

[32] Bolis G., Scarfone G., Raspagliesi F., Mangili G., Danese S., Scollo P., Russo D.L., Villa A., Aimone P.D., Scambia G. (2010). Paclitaxel/carboplatin versus topotecan/paclitaxel/carboplatin in patients with FIGO suboptimally resected stage III-IV epithelial ovarian cancer a multicenter, randomized study. Eur. J. Cancer.

[33] Tewari K.S., Burger R.A., Enserro D., Norquist B.M., Swisher E.M., Brady M.F., Bookman M.A., Fleming G.F., Huang H., Homesley H.D. (2019). Final Overall Survival of a Randomized Trial of Bevacizumab for Primary Treatment of Ovarian Cancer. J. Clin. Oncol..

[34] Monk B.J., Huang H.Q., Burger R.A., Mannel R.S., Homesley H.D., Fowler J., Greer B.E., Boente M., Liang S.X., Wenzel L. (2013). Patient reported outcomes of a randomized, placebo-controlled trial of bevacizumab in the front-line treatment of ovarian cancer: A Gynecologic Oncology Group Study. Gynecol. Oncol..

[35] Burger R.A., Brady M.F., Bookman M.A., Fleming G.F., Monk B.J., Huang H., Mannel R.S., Homesley H.D., Fowler J., Greer B.E. (2011). Incorporation of bevacizumab in the primary treatment of ovarian cancer. N. Engl. J. Med..

[36] González Martín A., Oza A.M., Embleton A.C., Pfisterer J., Ledermann J.A., Pujade-Lauraine E., Kristensen G., Bertrand M.A., Beale P., Cervantes A. (2019). Exploratory outcome analyses according to stage and/or residual disease in the ICON7 trial of carboplatin and paclitaxel with or without bevacizumab for newly diagnosed ovarian cancer. Gynecol. Oncol..

[37] Oza A.M., Cook A.D., Pfisterer J., Embleton A., Ledermann J.A., Pujade-Lauraine E., Kristensen G., Carey M.S., Beale P., Cervantes A. (2015). Standard chemotherapy with or without bevacizumab for women with newly diagnosed ovarian cancer (ICON7): Overall survival results of a phase 3 randomised trial. Lancet Oncol..

[38] Perren T.J., Swart A.M., Pfisterer J., Ledermann J.A., Pujade-Lauraine E., Kristensen G., Carey M.S., Beale P., Cervantes A., Kurzeder C. (2011). A Phase 3 Trial of Bevacizumab in Ovarian Cancer. N. Engl. J. Med..

[39] Coleman R.L., Fleming G.F., Brady M.F., Swisher E.M., Steffensen K.D., Friedlander M., Okamoto A., Moore K.N., Efrat Ben-Baruch N., Werner T.L. (2019). Veliparib with First-Line Chemotherapy and as Maintenance Therapy in Ovarian Cancer. N. Engl. J. Med..

[40] Wenzel L.B., Huang H.Q., Armstrong D.K., Walker J.L., Cella D., Mackey D. (2007). Health-related quality of life during and after intraperitoneal versus intravenous chemotherapy for optimally debulked ovarian cancer: A Gynecologic Oncology Group study. J. Clin. Oncol..

[41] Tan D.S., Kaye S.B. (2015). Chemotherapy for Patients with BRCA1 and BRCA2-Mutated Ovarian Cancer: Same or Different?. Am. Soc. Clin. Oncol. Educ. Book.

[42] WHO Classification of Tumours Editorial Board (2020). Female Genital Tumours: WHO Classification of Tumours.

